# Clinical factors affecting the rate of exodrift after surgery in patients with basic intermittent exotropia

**DOI:** 10.1038/s41598-021-86004-9

**Published:** 2021-03-22

**Authors:** Seungheon Kim, Suk-Gyu Ha, Young-Woo Suh, Seung-Hyun Kim

**Affiliations:** Department of Ophthalmology, Korea University, College of Medicine, Korea University Guro Hospital, 148, Gurodong-ro, Guro-gu, Seoul, 08308 Republic of Korea

**Keywords:** Diseases, Medical research, Risk factors

## Abstract

We investigated the period of postoperative exodrift during follow-up and clinical factors that affect the rate of exodrift after surgery in the patients with intermittent exotropia (IXT). A retrospective review of medical records of patients with exodrift who underwent bilateral rectus recession for IXT was performed. Exodrift was defined as angle of deviation greater than 10 prism diopters (PD) at distance and near. The median survival period of postoperative exodrift was analyzed using Kaplan Meier survival analysis. The patients were divided into two groups according to the median period of postoperative exodrift (early and late group). The weighted Cox’s proportional hazards regression analysis to investigate the risk factors that affect rate of postoperative exodrift was performed. A total of 108 patients was included. The preoperative angle of deviation at distance and near were 30.3 ± 7.2 PD and 29.5 ± 8.6 PD, respectively. The median survival period of postoperative exodrift was 24 months (range, 6–48 months).The angle of deviation at postoperative day 1 in early and late group were − 3.8 ± 5.5 PD (range, − 16–8 PD) and − 7.7 ± 4.6 PD (range, − 16–4 PD) (*p* < 0.01). Minus value means esodeviation. In regression analysis, the angle of deviation at postoperative day 1 was the significantly related with rate of exodrift (*p* < 0.01). The median period of exodrift after surgery was 24 months, angle of deviation at postoperative day 1 could affect the rate of exodrift in patients with IXT.

## Introduction

Patients with intermittent exotropia (IXT) who underwent surgery commonly develop exodrift over time^1^. The reported incidence of postoperative exodrift within 2 and 4 years after primary surgery is up to 42% and 50%, respectively^2^. Postoperative exodrift is largely responsible for the high recurrence and low success rate after surgery^3^. The rate at which the exodrift develops during the postoperative follow-up varies, with either relatively slow or fast progression^4,5^. It is challenging for a clinician to predict the time of onset and rate of progression of exodrift after surgery in IXT. There have been limited studies on predicting the onset of postoperative exodrift and investigating the relationship between clinical factors and the rate of postoperative exodrift^6^.

The purpose of this study is to investigate the median onset period of exodrift and the clinical factors which affect the rate at which exodrift develops after surgery in the patients with IXT.

## Materials and methods

This retrospective, single-center study followed the principles of the Declaration of Helsinki and was approved by the Institutional Review Board of Korea University Medicine. Written informed consent was obtained from all patients and their guardians. A medical chart review of patients with basic type of IXT who underwent bilateral lateral rectus recession between January 2007 and December 2014 was performed. All surgeries were performed under general anesthesia by one single experienced surgeon (S.H.K). Surgical amount was based on preoperative angle of deviation (Table 1)^7^. The patients had been followed at postoperative day 1, 1, 3, 6 months and 6 months interval. The patients who had exodrift during follow-up period after surgery were included in this study. The postoperative exodrift was defined as the angle of deviation greater than 10 prism diopters (PD) at distance and near during follow-up period. The angle of deviation (PD) was measured by alternate prism cover test at distance (6 m) and near (33 cm).

**Table 1:** Surgical amount in patients who underwent bilateral rectus recession.

Angle of deviation, prism diopter	Lateral rectus recession, mm
15	4
20	5
25	6
30	7
35	7.5
40	8
50	9

The following pre- and postoperative clinical data at every visits were reviewed: age at surgery, sex, refraction (diopter, D), distant suppression (Vectographic projector test; Vectogram, Reneau, France), inferior oblique overaction (IOOA), dissociated vertical deviation (DVD), angle of deviation at distance and near (PD) and near stereoacuity (Titmus stereo test, Stereo Optical Inc, Chicago, IL, USA). Refraction was calculated as spherical equivalent (D). Patients with other types of IXT such as convergence insufficiency or divergence excessive type, amblyopia, previous intraocular surgery or any neurological impairment, including cerebral palsy were excluded in this study.

The median survival period of exodrift after surgery was determined. The patients with exodrift were divided into two groups according to the median survival period of postoperative exodrift during follow-up.

Statistical analyses were performed with a statistical software program (StatLab, SPSS for Windows, version 21.0; SPSS, Inc., Chicago, IL). All continuous values were presented as mean ± standard deviation. Kaplan–Meier survival analysis was used to assess median survival period of exodrift after surgery. We performed Mann–Whitney test and Chi-square test to compare clinical data between two subgroups. We performed weighted Cox’s proportional hazards regression analysis to investigate the risk factors that affected exodrift after surgery. The p-values less than 0.05 were considered to be statistically significant.

## Results

Of the 662 patients who underwent surgery for the basic type of IXT, a total of 108 patients with postoperative exodrift was included in this study. The mean age at surgery was 5.2 ± 3.7 years (range, 1–36 years). Thirty one patients (28.7%) were male. There were 62 patients (57.4%) with preoperative distant suppression. There were 26 patients (24.1%) with IOOA or DVD. The mean preoperative angle of deviation was 30.3 ± 7.2 PD (range, 20 to 60 PD) at distance and 29.5 ± 8.6 PD (range, 10 to 60 PD) at near. Median stereoacuity at near was 60 arc of seconds (range, 60 to 100 arc of seconds). The mean postoperative follow-up period was 23.3 ± 12.1 months (range, 3–48 months). Ninety eight patients (91.6%) underwent reoperation for postoperative exodrift during follow-up period. The basic demographics were presented in Table 2.

**Table 2:** Basic demographics.

Patients	108
Age at surgery, years (range)	5.2 ± 3.7 (1–36)
Male (%)	31 (28.7)
**Refraction, D (range)**	
RE	− 0.10 ± 1.55 (− 5.0–3.50)
LE	− 0.10 ± 1.50 (− 4.75–4.50)
Distant suppression (%)	62 (57.4)
IOOA, DVD (%)	26 (24.1)
**Preoperative angle of deviation, PD (range)**	
Distance	30.3 ± 7.2 (20–60)
Near	29.5 ± 8.6 (10–60)
Median streoaucity, arc of second (IQR)	60 (60–100)
Postoperative follow up period, months (range)	22.3 ± 12.1 (6–48)

*D* diopters (calculated as spherical equivalent), *RE* right eye, *LE* left eye, *IOOA* inferiorobliqueoveraction, *DVD* dissociated vertical deviation, *PD* prism diopters, *IQR* interquatile range.

**Figure 1:**
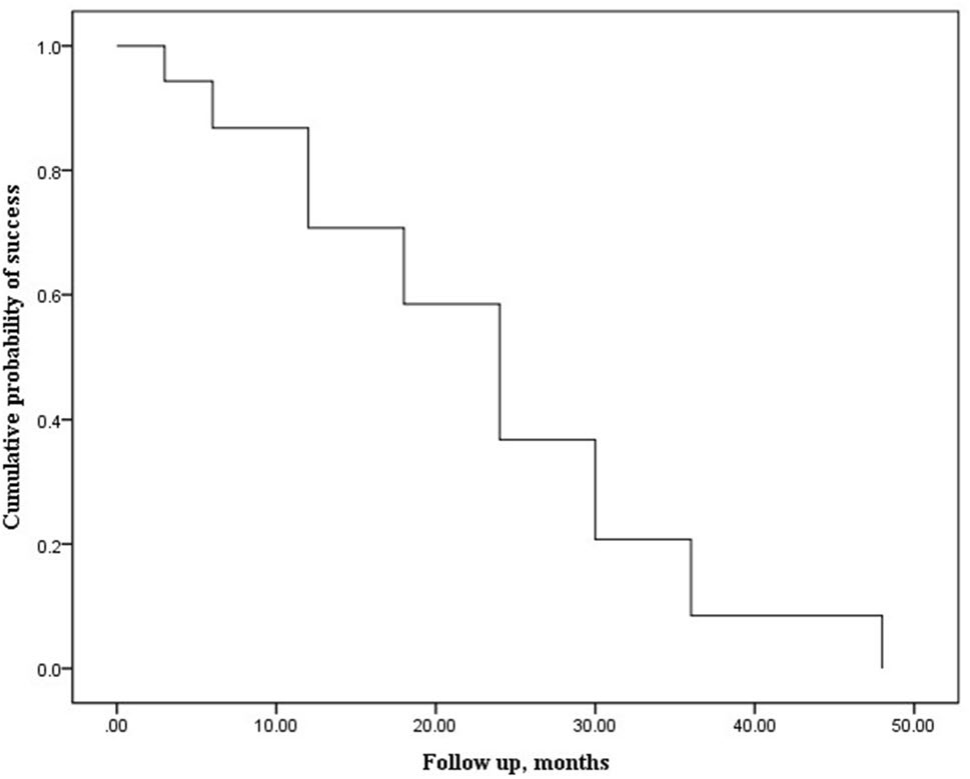
Kaplan–Meier survival analysis based on exodrift after surgery.

Using Kaplan–Meier survival analysis, median survival period was estimated during follow-up period (range, 6–48 months) as 24 months (Fig. 1). The patients were divided into two groups according to median survival period of exodrift after surgery, earlier (early group) or later (late group) than 24 months after surgery. The clinical data in two groups were showed in Table 3. The age at surgery, proportion of male and refraction were not significantly different between the two groups (*p* > 0.05, all). The distant suppression, IOOA or DVD were not significant different between two groups (*p* > 0.05, all). There were no significant differences of preoperative angle of deviation at distance and near in two groups (*p* = 0.93 and, 0.84). The angle of deviation at postoperative day 1 at distance and near in early group were − 3.8 ± 5.5 PD (range, − 16–8 PD) and − 2.9 ± 5.8 PD (range, − 18–10 PD), respectively. Minus value means esodeviation. The angle of deviations at postoperative day 1 at distance and near in late group were − 7.7 ± 4.6 PD (range, − 16–4 PD) and − 7.2 ± 4.7 PD (range, − 20–2 PD), respectively. The esodeviations at distance and near in exodrift in late group were greater than in early group (*p* < 0.01, all).

**Table 3:** Comparison of clinical characteristics between subgroups.

	Early (≤ 24 months)	Late (> 24 months)	*p*
Patients	67	41	–
Age at primary operation, years (range)	5.5 ± 4.9 (1–36)	5.0 ± 1.8 (2–12)	0.45*
Male (%)	21 (31.3)	9 (23.1)	0.25^a^
**Refraction, D (range)**			
RE	− 0.20 ± 1.72 (− 5.00–3.50)	0.09 ± 1.25 (− 2.50–2.25)	0.54*
LE	− 0.17 ± 1.77 (− 4.75–4.50)	0.13 ± 1.17 (− 3.0–2.2.5)	0.35*
Distant suppression (%)	38 (56.7)	24 (58.5)	0.44^a^
IOOA/DVD (%)	18 (26.5)	8 (19.5)	0.28^a^
**Preoperative angle of deviation, PD (range)**			
Distance	30.2 ± 1.7 (20–60)	30.0 ± 7.3 (20–60)	0.93*
Near	29.9 ± 8.7 (12–60)	29.0 ± 8.7 (10–60)	0.84*
**Postoperative angle of deivation at POD1, PD (range)**			
Distance	− 3.8 ± 5.5 (− 16–8)**	− 7.7 ± 4.6 (− 16–4)	< 0.01*
Near	− 2.2 ± 5.8 (− 18–10)	− 7.2 ± 4.7 (− 20–2)	< 0.01*
Median stereoacuity, arc of seconds (IQR)	60 (60–100)	60 (60–100)	0.45

*D* diopters (calculated as spherical equivalent), *RE* right eye, *LE* left eye, *IOOA* inferiorobliqueoveraction, *DVD* dissociated vertical deviation, *PD* prism diopters, *POD* postoperative day, *IQR* interquatile range.

*Mann–whitney test, **minus value means esodeviation.

^a^Chi-square test.

In weighted Cox’s proportional hazards regression analysis, a postoperative angle of deviation at distance (HR = 1.09, CI = 1.04–1.12, *p* < 0.01) and near (HR = 1.09, CI = 1.04–1.1, *p* < 0.01) were significant related to the occurrence of postoperative exodrift (Table 4).

**Table 4:** Weighted Cox’s proportional hazard model regression analysis based on period of exodrift after surgery.

	HR	95% CI	*p* value
Age at surgery	1.02	0.11–9.86	0.065
Male	0.804	0.56–1.23	0.316
**Refraction**			
RE	0.949	0.83–1.19	0.451
LE	0.949	0.82–1.10	0.469
Distant suppression	0.896	0.56–1.42	0.64
IOOA, DVD	1.12	0.69–1.71	0.74
**Preoperative angle of deviation**			
Distance	1.01	0.98–1.03	0.85
Near	1.01	0.98–1.02	0.91
**Postoperative esodeviation at POD1**			
Distance	1.09	1.04–1.12	< 0.01
Near	1.09	1.04–1.13	< 0.01
Median stereoacuity	0.99	0.98–1.01	0.56

*RE* right eye, *LE* left eye, *IOOA* inferiorobliqueoveraction, *DVD* dissociated vertical deviation, *POD* postoperative day.

## Discussion

Our study showed that the median time of onset of exodrift after surgery was 24 months during follow-up period and the initial postoperative angle of deviation was related to the rate of postoperative exodrift.

Clinical factors such as the preoperative angle of deviation, distance—near disparity, age at surgery, refractive error, and surgical methods have been reported as predictors of surgical outcome in IXT^8–10^.

The exact duration of follow-up period during the surgeon should observe the patients remains unclear. Kwon and Kim reported that stabilization of the postoperative angle of deviation was achieved at three years after surgery in patients with IXT^11^. However, there have been a few previous studies where the occurrence of exodrift was prevalent after surgery in IXT^11,12^. In our study, the median onset of exodrift period after surgery was 24 months, but ranging from 3 to 48 months. Thus, it may be clinically helpful for the surgeon to observe the patient for least 24 months after surgery for IXT.

In study by Park and Kim, exodrift rates over a follow-up period of 12 months were reported. They indicated that the exodrift rate was fastest at postoperative weeks 1–3, and showed the strongest correlation with overall drift rate^3^. Other previous studies have also shown that more than half of patients with postoperative exodrift will develop it during the first postoperative year^11,12^. Yam et al. reported in a large cohort study that the proportion of patients with postoperative exodrift increased from 62% at 6 weeks to 84% at 3 years and that a larger preoperative deviation is associated with both a larger early and late postoperative drift^13^. Kim and Kim reported that early onset exodrift after the first surgery appeared to be a predictor of recurrent exodrift, however, no significant difference in surgical outcome was observed after the second surgery^6^.

In this study, clinical factors such as the age at surgery, sex, refraction and preoperative angle of deviation at distance and near were not significantly different between early and late group. However, the initial postoperative angle of deviation at distance and near were related to the rate of exodrift after surgery.

In previous reports, many authors have demonstrated that an initial overcorrection after surgery is associated with favorable long-term motor alignment in patients with IXT and recurrent exotropia^14–17^. However, Yam et al. reported that the exodrift in the initial overcorrection group was significantly greater than that in initial undercorrection group at 6 weeks and 3 years postoperatively^13^. In our study, initial overcorrection at postoperative day 1 in late group was greater than in early group and postoperative angle of deviation had a significant relation to the rate of exodrift during follow-up period.

There were some limitations in this study. First, the study was limited by its retrospective nature. Second, the patients in this study underwent only bilateral rectus recession, so we were unable to compare the results of different other surgical methods. Different surgical methods may affect the surgical outcome and prevalence of postoperative exodrift. Third, it was difficult to determine the exact amount of initial postoperative overcorrection which could affect the development of postoperative exodrift. Nevertheless, our study had a large sample group of patients with basic IXT that underwent bilateral rectus recession with a long follow-up period.

In conclusion, the median onset period of exodrift after primary surgery was 24 months and the initial postoperative angle of deviation affected the rate of progression of exodrift in patients with IXT.
